# Scientific validation of three-dimensional stereophotogrammetry compared to the IGAIS clinical scale for assessing wrinkles and scars after laser treatment

**DOI:** 10.1038/s41598-021-91922-9

**Published:** 2021-06-11

**Authors:** Barbara Helena Barcaro Machado, Ivy Dantas De Melo E. Silva, Walter Marou Pautrat, James Frame, Mohammad Najlah

**Affiliations:** 1 Pharmaceutical Research Group, School of Allied Health, Faculty of Health, Education, Medicine and Social Care, Anglia Ruskin University, Bishops Hall Lane, Chelmsford, CM1 1SQ UK; 2 Hospital Municipal Nossa Senhora do Loreto, Rio de Janeiro, Brazil; 3 Universidad Cientifica del Sur, Lima, Peru; 4 The School of Medicine, Faculty of Health, Education, Medicine and Social Care, Chelmsford Campus, Bishop Hall Lane, Chelmsford, CM1 1SQ UK

**Keywords:** Biological techniques, Biotechnology, Computational biology and bioinformatics, Medical research

## Abstract

Measuring outcomes from treatments to the skin is either reliant upon patient’s subjective feedback or scale-based peer assessments. Three-Dimensional stereophotogrammetry intend to accurately quantify skin microtopography before and after treatments. The objective of this study is comparing the accuracy of stereophotogrammetry with a scale-based peer evaluation in assessing topographical changes to skin surface following laser treatment. A 3D stereophotogrammetry system photographed skin surface of 48 patients with facial wrinkles or scars before and three months after laser resurfacing, followed immediately by topical application of vitamin C. The software measured changes in skin roughness, wrinkle depth and scar volume. Images were presented to three observers, each independently scoring cutaneous improvement according to Investigator Global Aesthetic Improvement Scale (IGAIS). As for the results, a trend reflecting skin/scar improvement was reported by 3D SPM measurements and raters. The percentage of topographical change given by the raters matched 3D SPM findings. Agreement was highest when observers analysed 3D images. However, observers overestimated skin improvement in a nontreatment control whilst 3D SPM was precise in detecting absence of intervention. This study confirmed a direct correlation between the IGAIS clinical scale and 3D SPM and confirmed the efficacy and accuracy of the latter when assessing cutaneous microtopography alterations as a response to laser treatment.

## Introduction

Many validated investigative tools for assessment of skin relief in response to topical therapies are inadequate or are too inaccurate to quantify microtopographical changes^1–33^. This is largely because skin is neither a linear nor a bi-planar structure. In addition, there is an innate inability to precisely measure changes in response to a surface treatment. Two-dimensional photograph-based analyses by observers are vulnerable to subjective criticism, variable magnifications, backgrounds and postures.

The ideal quantitative assessment is a three-dimensional evaluation within a standard-setting^14, 26^ and Areal Topography (AT), based on a pair of two-dimensional (2D) maps, which can delineate the shape and features of such surfaces. AT combines photographic documentation and uses algorithms that capture and provide precise 3D information on surfaces and textures^34^. Three-dimensional stereophotogrammetry (3D SPM) is an imaging system based on AT. This method provides information on a surface by crossing data obtained from a pair of slightly different stereoscopic two-dimensional pictures from two different angles^30, 35^. The images are processed by the mathematical algorithms embedded in a software performing a spatial analysis based on the intersection of ray bundles derived from both photographs. The software detects and quantifies subtle differences in height, depth, width and texture of a surface^24, 34^. Three-dimensional stereophotogrammetry has been validated as a precise, harmless, non-invasive system to monitor surface irregularities including cutaneous tumors, scars and wrinkles^1–3, 21–33, 36^. However, most studies lack robust statistical analysis or have not included objective documentation regarding pre and post-procedure comparison.

The aim of this study is to validate 3D SPM as an objective tool for dermatographic assessment against the subjective scoring of three plastic surgeons with experience in laser skin resurfacing. We hypothesised that both methods would agree and produce similar assessment. Primary outcome was to compare the clinical assessment of three observers, experts in the field, based on a clinical scale (IGAIS scoring system) with measurements obtained by the 3D SPM system. These measurements concerned the topographical changes to the surface contour of skin wrinkles and hypertrophic scars after laser resurfacing and application of Vitamin C. The results obtained through both methods were statistically assessed and compared.

## Material and methods

This study has obtained ethical approval by Associação Congregação Santa Catarina (Brazil) and Anglia Ruskin University (UK) (approved on 5th September 2017). The clinical trial is registered at Plataforma Brazil under the number: 71398617.7.0000.5664 (registration on 18th July 2017) and REBEC, participant of the WHO International Clinical Trial Registry Platform (UTN number U1111-1262-9267).

The sample size calculation for the present study was based on a pilot study as part of PhD research of the first author and established that the minimal *n* for statistical significance was 44 patients. A total of 48 female patients aged between 23 and 70 years old with Fitzpatrick skin type I to IV were recruited in Rio de Janeiro between September and November 2017. All patients consented to participate and signed an informed consent form that among other authorizations also allowed the use of images in medical publications, including online open-access journals. Twenty-two patients with visible and hypertrophic scars comprised group DS (45.8%), and twenty-six patients with facial wrinkles comprised group R (54.2%). Patients in Group DS had hypertrophic scars on the abdomen (7 patients), on the face (8 patients) and limbs (7 patients). The mean age of the scars was 35.7 ± 72.9 months. Patients who could not be available for a 3-month follow-up assessment and those having received treatments in the area to be addressed up to 6 months before this study, were excluded.

A contactless 3D SPM system (LifeViz Micro, QuantifiCare, France) was used by the first author to photograph the wrinkle and scar surface according to the study group. Photographs were uploaded to a computer and the software Dermapix was used to objectively measure any changes in skin topography, following laser skin resurfacing and application of surface vitamin C, to patients in each group.

All patients in both groups were instructed to remove surface cosmetics and topical medication from the defined areas prior to investigation. They were placed on a printed protractor scale laid on the floor, and the camera was positioned perpendicularly to the skin so that pre and post-procedure images could present identical main axis, angle and focus. The anatomical reference points were individually determined by a laser tape measure.

After photographic documentation, a topical anaesthetic ointment containing lidocaine 7% was applied on the skin for thirty minutes before the procedure. The skin was cleaned, and the patient was treated. The same laser protocol was applied to all patients in both study groups R and DS and included four passes with a 2940 nm erbium:Yag ablative laser resurfacing (Starlux 500 Palomar Inc.). The laser energy output delivered by the blue optic 6 × 6 mm handpiece was 9 mJ/μb of short pulse energy (250 ms) and of 8 mJ/μb of the long pulse (5 ms). After the laser treatment, 200 mg of vitamin C (ascorbic acid—Vitasantisa®) was applied on the skin surface and kept under occlusion and protected from light exposure for 30 min.

Patients were followed-up approximately 90 days after the procedure when post-procedure photographs were obtained. The pre and post-procedure 5.25 cm × 7 cm pictures were transferred to the software Dermapix and rearranged in individual files. They were precisely overlapped in a process called synchronization by which landmarks are established in both pre and post-procedure photographs. All images that did not meet this criterion were discarded.

The software permitted the selection of a three-dimensional reconstruction tool which displayed a 3D image on the computer screen that reflected the skin surface. After this 3D reconstruction process, a coefficient called “Sigma” was automatically exhibited. Sigma is a plane surface reference from which elevations (positive volumes) or demotions (negative volumes) can be detected and quantified. Sigma varies from 1 to 99, and its value is restrained relevant to the researcher’s goal. A Sigma value of 10 was applied to all cases because it captured the subtle changes in cutaneous microtopography required in this study.

The software Dermapix was used to design a contour encompassing the treatment area and a small amount of normal surrounding skin, to allow for some shrinkage or stretching that can accrue from laser resurfacing treatments. As the photographs were synchronised, the software replicated the same electronic marking to the post-treatment image and automatically calculated information on the skin surface^21, 28^, volume^16, 28, 31^, roughness^5, 16, 18–20, 24, 34^, average height^16^ and average depth^25^. Roughness is defined as the arithmetic mean of peak-to-valley-height skin characteristics^19, 34, 37^ and is specified by the International Organization for Standardization (ISO 3274:’96 and ISO 4287:’97). Its change is linked to the ageing process, scars, after-treatment alteration, and some pathological conditions^36, 38–43^. The facial wrinkle (perioral or periorbital) was analysed using roughness and the average depth of the wrinkles as volume is not suitable for analysing this skin alteration. For scars, volume and roughness were quantified as the average depth is not a feature usually involved in hypertrophic scars.

A set of pre and post‐treatment two-dimensional and three-dimensional digital images of the 48 patients were arranged as a slide presentation and assessed by the observers. After receiving clear and simple guidelines, they employed the visual analogue Investigator Global Aesthetic Improvement Scale (IGAIS)^4, 6, 11, 44^ to independently rate the post-procedure changes to surface topography within an area highlighted with a black marker. IGAIS establishes score 0 for null or minimal change (0–25% of skin improvement), 1 for mild improvement (25–50%), 2 for moderate improvement (50–75%) and 3 for significant improvement (> 75%). In group R, Fitzpatrick grade 1 shallow visible wrinkle (less than 1 mm in depth) up to Fitzpatrick grade 3 prominent deep furrow wrinkles (3 mm or more in depth) were analysed. In the group DS, the pre-treatment scars area exhibiting the most prominent irregularity (scar elevation) were compared. We constrained the area under investigation so that the three assessors could focus their observation to the same area measured by 3D SPM.

The quantitative data provided by the software was based on skin characteristics and is always the same, regardless of visualising 2D or 3D images. On the other hand, as clinical observation is based on visual perception of the deformity, the observers rated the skin alteration based on the observation of 2D photographs and 3D images. The experts also estimated the percentage of modification for the delimited skin areas based on the 3D images. Finally, they answered two questions: (a) Is the scale sufficient to quantify the volumetric change or the skin relief alteration after the procedure? (b) Do the 3D images improve the ability to use the scale to quantify the skin alteration after the procedure?

The 3D SPM data output was compared with the IGAIS scoring system. One patient was randomly selected to be a negative control (non-treatment), and both the observers and 3D SPM were blinded to this information. Two patients who produced negative value outcomes were not excluded.

Statistical evaluation was performed using SPSS statistical package version 24 (IBM Inc., Chicago, USA). Shapiro–Wilk test and histograms verified that the numerical variables provided by 3D SPM (skin roughness, scar volume and wrinkle average depth) were not normally distributed. Data were displayed as median, mean, SD and Interquartile Interval (IQI) which represents the range between the 25th and 75th percentiles. Table 1 is a summary of the material and statistical tests used in this study.

**Table 1:** Summary of the material and statistical tests involved in this study.

Recruitment age	Group DS: 23–70 years old	Group R: 49–70 years old	
Number of participants (*n*)	Group DS: 22	Group R: 26	
Anatomical distribution of scars—group DS	Abdomen: 7	Face: 8	Limbs: 7
Wrinkle location—group R	Perioral: 9	Periorbital: 17	
Parameters analysed	Group DS: scar roughness, scar volume	Group R: Skin roughness, wrinkle average depth	
Statistical tests (CI 95%)	Purpose		
Shapiro–Wilk and histograms	Verify the data distribution of the variables provided by the 3D SPM system (skin roughness, scar volume, scar roughness and wrinkle average depth)		
Wilcoxon signed rank-test	Verify the statistical significance of variation in roughness, scar volume and wrinkle average depth		
Mann Whitney	Analise the median of the percentage of skin improvement provided by the clinical observers for both study groups		
ICC	Investigate IGAIS for homogeneity and internal consistency. Interpretation^45^: ICC < 0.4 = poor reliability ICC 0.41–0.74 = moderate reliability ICC ≥ 0.75 = excellent reliability		
Spearman Rho	Measure the association between IGAIS and 3D SPM (based on scores provided by IGAIS) Interpretation: Rho up to ± 0.3 = negligible correlation Rho ± 0.31–0.5 = low correlation Rho ± 0.51–0.7 = moderate correlation Rho 0.71–0.9 = high correlation Rho > ± 0.9 = very high correlation		
Spearman Rho	Measure the association between IGAIS and 3D SPM (based on the percentage of skin modification)		
Kappa coefficient	Measure the interrater agreement Interpretation^46^:2 Kappa ≤ 0.19 = no agreement Kappa 0.2–0.39 = poor agreement Kappa 0.4–0.59 = moderate agreement Kappa 0.6–0.79 = good agreement Kappa ≥ 0.8 = very good/excellent agreement		
Bland–Altman plots	Investigate the agreement between both methods (IGAIS and 3D SPM)		

A positive post-treatment variation was expressed by the numeric reduction of values concerning the variables. Conversely, the increase of post-treatment measurements corresponded to a negative response to the treatment. Variation between the pre and post-treatment skin roughness (Rgh), scar volume (V_DS_) and wrinkle average depth (AD_R_) was calculated in terms of percentage (∂ reduction) based on the formula:

$${\text{Percentage}}\;{\text{of}}\;{\text{parameter}}\;{\text{reduction}}\left( {\partial \;{\text{reduction}}} \right) = \left( {{\text{pre}} - {\text{post}}\;{\text{measure}}} \right) \div {\text{pre}}\;{\text{measure}} \times 100$$

Wilcoxon signed-rank test was applied to verify the statistical significance of the variation of the parameters measured by the 3D SPM system. Mann–Whitney test was run to establish whether there was statistical significance concerning the variables under analysis (median of the percentage of skin improvement provided by the clinical observers).

The intra-class correlation coefficient (ICC—95% CI, two-way model and based on consistency) was used to investigate the interrater reliability, i.e. the scale homogeneity and consistency. As clinical observation is subjective to individual variability, the consistency variability established if the clinical observations received the same relative ranking. Reliability value ranges between o and 1, with values closer to 1 representing stronger reliability. As there is lack of standard for reporting ICC, the interpretation of ICC values followed Shrout & Fleiss^45^, who described that values less than 0.4 can be considered poor reliability, values between 0.41 and 0.74 indicate moderate reliability and values greater than 0.75 are indicative of excellent reliability (Table 1).

Spearman’s *rho* measured the association between both methods and Kappa coefficient measured the correlation between each pair of observers and the 3D SPM system in relation to the total sample^46^. To permit this calculation, the simultaneous computation of ∂ reduction of both variables measured in each study group was calculated by the aforementioned formula (Rgh_DS_ ∂ reduction plus V_DS_ ∂ reduction in group DS, and Rgh_R_ ∂ reduction plus AD_R_ ∂ reduction in group R). The computation was transformed into ordinal data, based on the same IGAIS intervals described before. Then these ordinal data were compared with the scores provided by the observers.

According to Landis & Koch^46^, Kappa values ≤ 0.19 represent no agreement, values between 0.2 and 0.39 demonstrate poor agreement, 0.4 to 0.59 indicate moderate agreement, 0.6 to 0.79 represent good agreement and Kappa ≥ 0.8 indicate very/excellent agreement. As for Spearman correlation, rho values vary from 1.0 to -1.0. The stronger the correlation, the closer the correlation coefficient comes to ± 1 (Table 1).

Finally, the percentage of skin improvement provided by the observers based on the 3D images and the data delivered by the 3D SPM system were analysed through Spearman’s rho and Bland–Altman plots. These plots do not evaluate correlation, but the agreement between two methods (the clinical observation based on IGAIS and the 3D SPM system) that measure the same quantity. Bland–Altman plots include limits of agreement and confidence intervals to establish whether the limits are acceptable differences from a clinical point of view.

The criterion to determine significance was alpha level 0.05 and Confidence Interval (CI) of 95% in all statistical tests.

### Ethics approval

This research was approved by Association Congregation of Santa Catarina Ethics Committee and Plataforma Brazil (registration number) CAAE:71,398,617.7.0000.5664, and the Faculty Research Ethics Panel (FREP) at Anglia Ruskin University. The clinical study took place in Rio de Janeiro, Brazil, and complied with the principles of the World Medical Association Declaration of Helsinki (2013). All patients signed a consent form which was revised by both Ethics committees.

### Consent to participate

All patients have consented to participate and to and have signed an informed consent form that has been submitted to both Ethics committees. Participants signed the informed consent form in agreement with the use of photographs of their skin in publications.

### Consent for publication

The photographs are property of BHBM (the first author) and can be published.

## Results

The final assessments of patients in both R and DS groups after laser skin surface ablation and application of vitamin C was at three months (mean 91.9 days ± 4.6 SD). For the total sample, mean age was 53.6 years old (± 13.5 SD). Participants in group R were significantly older (p < 0.05) with age ranging from 49 to 70 years (mean 61.8 years old ± 6.1 years. The mean age of patients in group DS was of 43.9 ± 13.3 years old. The majority of subjects presented with Fitzpatrick skin type II (34%) and III (39.6%). An overall positive skin change was detected in 45 patients (93.75%). In two patients (4.1% of the cases), the scars worsened.

Based on the 3D SPM findings, the non-parametric Wilcoxon signed-rank test confirmed a statistically significant variation (reduction) in roughness measurements (*p* < 0.01). There was an important reduction in the average depth measurement in group R (*p* < 0.01). Conversely, no significant variation in volume measurement in group DS was observed (*p* = 0.37).

**Figure 1:**
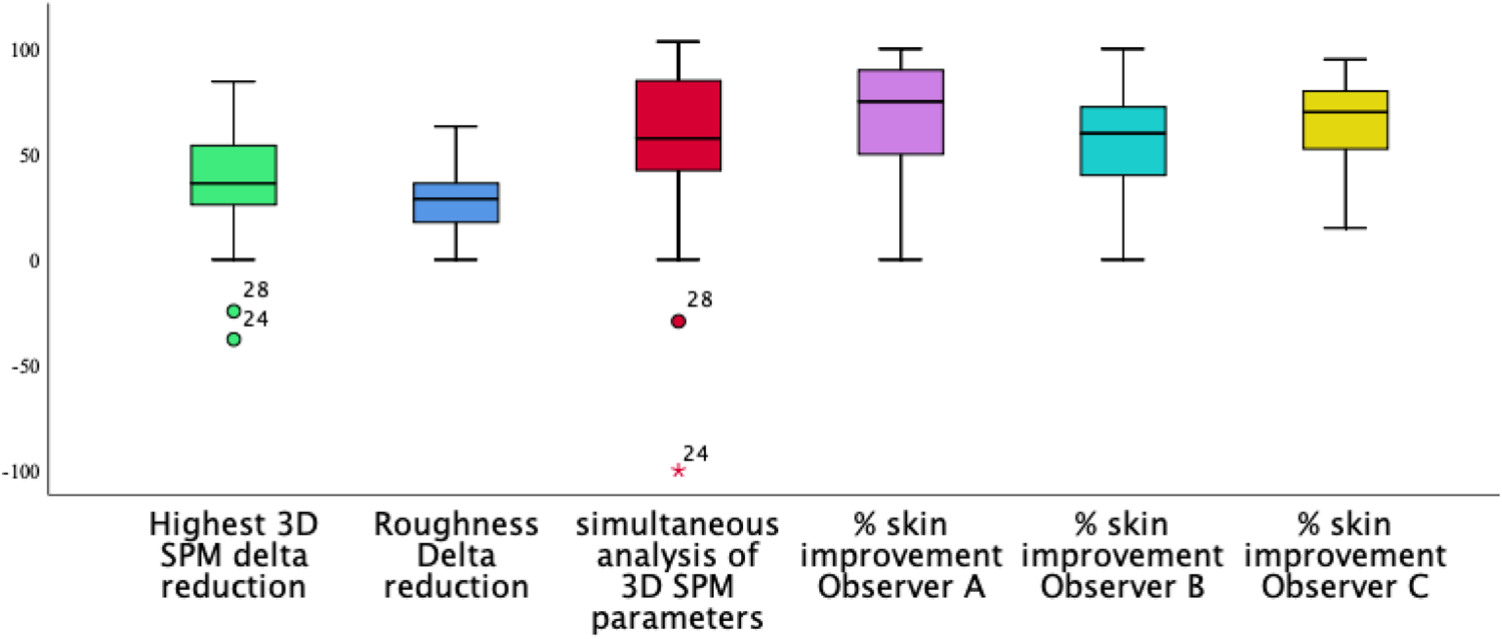
Graphic illustration representing the percentage of skin modification (∂ reduction) according to the observers (**A**,**B**,**C**) and the 3D SPM readouts (the highest ∂ reduction and roughness ∂ reduction in the total sample, and the simultaneous computation of RghDS ∂ reduction plus VDS ∂ reduction in group DS, and RghR ∂ reduction plus ADR ∂ reduction in group R).

Figure 1 is the graphic illustration of the estimated percentage of skin improvement according to each observer, the roughness ∂ reduction in the total sample, and the highest ∂ reduction (related to the parameter that presented the highest percentage of skin modification), and the simultaneous computation of Rgh_DS_ ∂ reduction plus V_DS_ ∂ reduction in group DS (as volume and scar roughness were evaluated), and Rgh_R_ ∂ reduction plus AD_R_ ∂ reduction in group R (as skin roughness and the average depth of the wrinkle were analysed). According to the boxplot, the simultaneous computation of both parameters had a graphic similar to the percentage of skin improvement provided by the observers.

Mann–Whitney test applied to the median of the estimated percentage of skin improvement demonstrated that all observers provided a higher percentage for skin modification in group R compared to scar alteration in group DS. The ∂ reduction based on 3D SPM readouts was lower (Table 2).

**Table 2:** Mann–Whitney test analysing the estimated percentage of skin improvement provided by the observers and 3D SPM objective data. Results by group.

Variable	Group R (Wrinkle) (*n* = 26)		Group DS (Scar) (*n* = 22)		*p-*value
	Median	IQI	Median	IQI	
Observer A % of improvement	90.0	58.8–100	67.5	36.3–80.0	**0.010**
Observer B % of improvement	60.0	48.8–75.8	57.5	25.8–71.3	0.28
Observer C % of improvement	80.0	63.8–85.0	57.5	35.0–80.0	**0.010**
3D SPM—volume ∂ reduction	N/A	N/A	26.2	10.0–39.3	N/A
3D SPM—average depth ∂ reduction	33.3	22.1–54.0	N/A	N/A	N/A
3D SPM—roughness ∂ reduction	28.4	19.1–33.3	28.8	10.8–42.7	0.75
3D SPM—highest ∂ reduction	39.8	28.7–54.0	40.2	25–56.0	0.91
3D SPM—simultaneous analysis of both parameters	62.6	46.1–87.5	48.0	33.1–74.4	0.10

*IQI* interquartile interval (25th percentile–75th percentile), *N/A* non-applicable.

The intraclass correlation coefficient (ICC) investigated IGAIS for homogeneity and consistency. The statistically significant ICC (*p* < 0.01) confirmed the reliability of using IGAIS. ICC based on the analysis of 3D images was excellent according to Sprout and Fleiss^45^ compared with the ICC based on the observation of 2D images (Table 3).

**Table 3:** Intraclass Correlation Coefficient for internal consistency and the descriptive level (*p*-value) of skin improvement among the observers and the 3D SPM (total sample *n* = 48).

Concordance			ICC	95% CI		*p* value
**2 d images**						
Observer A	x	Observer B	0.72	0.50	0.84	< 0.001
Observer A	x	Observer C	0.62	0.27	0.79	< 0.001
Observer B	x	Observer C	0.78	0.61	0.88	< 0.001
A x B x C			0.79	0.65	0.87	< 0.001
**3 d images**						
Observer A	x	Observer B	0.85	0.74	0.92	< 0.001
Observer A	x	Observer C	0.78	0.52	0.89	< 0.001
Observer B	x	Observer C	0.83	0.60	0.92	< 0.001
A x B x C			0.88	0.78	0.93	< 0.001

Two-way Model; 95% CI (confidence interval); x = versus.

As mentioned before, the ∂ reduction concerning each variable was transformed into ordinal variables, based on the same IGAIS interval to be compared with the scores provided by the observers. Spearman’s *rho* measured the association and Kappa coefficient measured the agreement between both methods (the clinical scale and the 3D SPM system) in relation to the total sample^45^. The agreement was higher when the observers analysed the 3D images (Table 4). Both coefficients confirmed that the agreement between observer A and 3D SPM was high, whether by comparing the scores representing the highest ∂ reduction detected by the software (*rho* = 1 and Kappa = 1; *p* < 0.01) or by comparing the scores related to the simultaneous computation of both ∂ reduction parameters, according to the study group (*rho* = 0.99 and Kappa 0.97; *p* < 0.01). The agreement between each observer and the roughness ∂ reduction was weaker and not statistically significant.

**Table 4:** Spearman’s *rho* correlation coefficient, the Kappa statistics and the descriptive level (*p*-value) based on the ordinal data (scores).

	Spearman’s *rho*	*p-*value	Kappa	*p-*value
**Observers scores on 2D images (*n* = 48 cases)**				
Observer A x Observer B	0.52	< 0.001	0.41	< 0.001
Observer A x Observer C	0.54	< 0.001	0.16	0.59
Observer A x 3D SPM roughness ∂ reduction	0.19	0.204	0.06	0.13
Observer A x 3D SPM highest ∂ reduction	0.45	< 0.001	0.18	0.03
Observer A x 3D SPM simultaneous analysis of both parameters	0.47	< 0.001	0.18	0.35
Observer B x Observer C	0.67	< 0.001	0.38	< 0.001
Observer B x 3D SPM roughness ∂ reduction	0.37	< 0.001	0.10	0.04
Observer B x 3D SPM highest ∂ reduction	0.48	< 0.001	0.18	0.03
Observer B x 3D SPM simultaneous analysis of both parameters	0.48	< 0.001	0.25	0.003
Observer C x 3D SPM roughness ∂ reduction	0.61	< 0.001	0.13	0.82
Observer C x 3D SPM highest ∂ reduction	0.70	< 0.001	0.41	< 0.001
Observer C x 3D SPM simultaneous analysis of both parameters	0.70	< 0.001	0.43	< 0.001
**Observers scores on 3D images (*n* = 48 cases)**				
Observer A x Observer B	0.75	< 0.001	0.46	< 0.001
Observer A x Observer C	0.67	< 0.001	0.97	< 0.001
Observer A x 3D SPM roughness ∂ reduction	0.51	< 0.001	0.13	0.034
Observer A x 3D SPM highest ∂ reduction	**1.0**	< 0.001	**1.0**	< 0.001
Observer A x 3D SPM simultaneous analysis both parameters	**0.99**	< 0.001	**0.97**	< 0.001
Observer B x Observer C	0.74	< 0.001	0.49	< 0.001
Observer B x 3D SPM roughness ∂ reduction	0.47	0.01	−0.01	0.815
Observer B x 3D SPM highest ∂ reduction	0.75	< 0.001	0.46	< 0.001
Observer B x 3D SPM simultaneous analysis of both parameters	0.79	< 0.001	0.46	< 0.001
Observer C x 3D SPM roughness ∂ reduction	0.52	< 0.001	0.01	0.98
Observer C x 3D SPM highest ∂ reduction	0.67	< 0.001	0.36	< 0.001
Observer C x 3D SPM simultaneous analysis of both parameters	0.69	< 0.001	0.36	< 0.001

x = versus.

The percentage of skin improvement provided by the observers based on the 3D images was compared to each other and to the data delivered by the 3D SPM system (Table 5). Spearman’s *rho* was higher when the highest ∂ reduction, and the simultaneous computation of Rgh_DS_ + V_DS_ ∂ reduction in group DS and Rgh_R_ + AD_R_ ∂ reduction in group R were compared to the percentage provided by each observer (*p* < 0.01). The best agreement was between Observers A and C (0.843).

**Table 5:** Spearman’s *rho* correlation coefficient and the descriptive level (*p*-value) of skin modification based on the percentages provided by the observers and 3D SPM (*n* = 48 cases).

Observer	Versus	Observers percentages on 3D	*rho*	*p-*value
Observer A	x	Observer B	0.754	< 0.001
Observer A	x	Observer C	**0.843**	< 0.001
Observer A	x	3D SPM roughness ∂ reduction	0.493	< 0.001
Observer A	x	3D SPM highest ∂ reduction	0.620	< 0.001
Observer A	x	3D SPM simultaneous analysis both parameters	**0.652**	< 0.001
Observer B	x	Observer C	0.781	< 0.001
Observer B	x	3D SPM roughness ∂ reduction	0.430	< 0.001
Observer B	x	3D SPM highest ∂ reduction	0.654	< 0.001
Observer B	x	3D SPM simultaneous analysis both parameters	**0.682**	< 0.001
Observer C	x	3D SPM roughness ∂ reduction	0.361	0.002
Observer C	x	3D SPM highest ∂ reduction	0.621	< 0.001
Observer C	x	3D SPM simultaneous analysis both parameters	**0.699**	< 0.001

**Figure 2:**
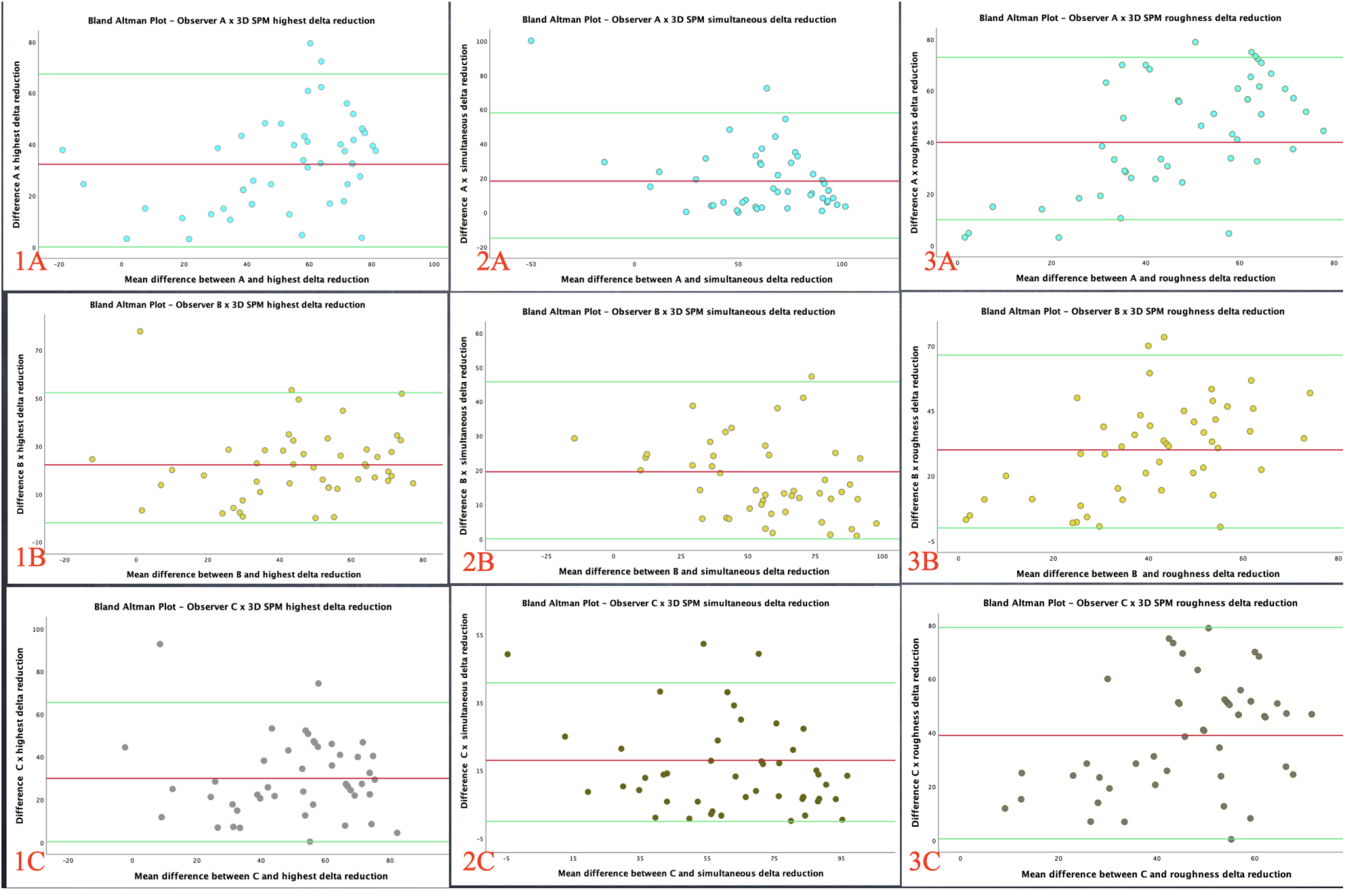
Bland–Altman plots. The letters correspond to observers (**A**,**B**,**C**) column 1 is the highest ∂ reduction, column 2 is the simultaneous computation of the two parameters analysed in each group, and column 3 represents the roughness delta reduction. The green lines are the limits of agreement and confidence intervals. The few points outside the limits of agreement confirmed the concordance among observers and the 3D SPM system.

Bland–Altman plots considering the total sample (48 patients) were applied to the numerical data to further investigate the agreement between both methods, the clinical observation based on IGAIS and the 3D SPM system (Fig. 2). These plots represent the dispersion of the differences between the estimated percentage of modification provided by each observer in relation to the 3D SPM ∂ reduction readouts against their respective average. Concordance was higher between each observer and the highest ∂ reduction (column 1) followed by the simultaneous computation of the two parameters analysed in each group (column 2). Column 3 represents the roughness delta reduction, the parameter measured in both study groups. Through the amplitude of the concordance intervals, the quality of agreement can be determined, and biases can be detected. Despite the moderately wide intervals, the random distribution of differences over the mean values confirmed the absence of systematic behavior.

**Figure 3:**
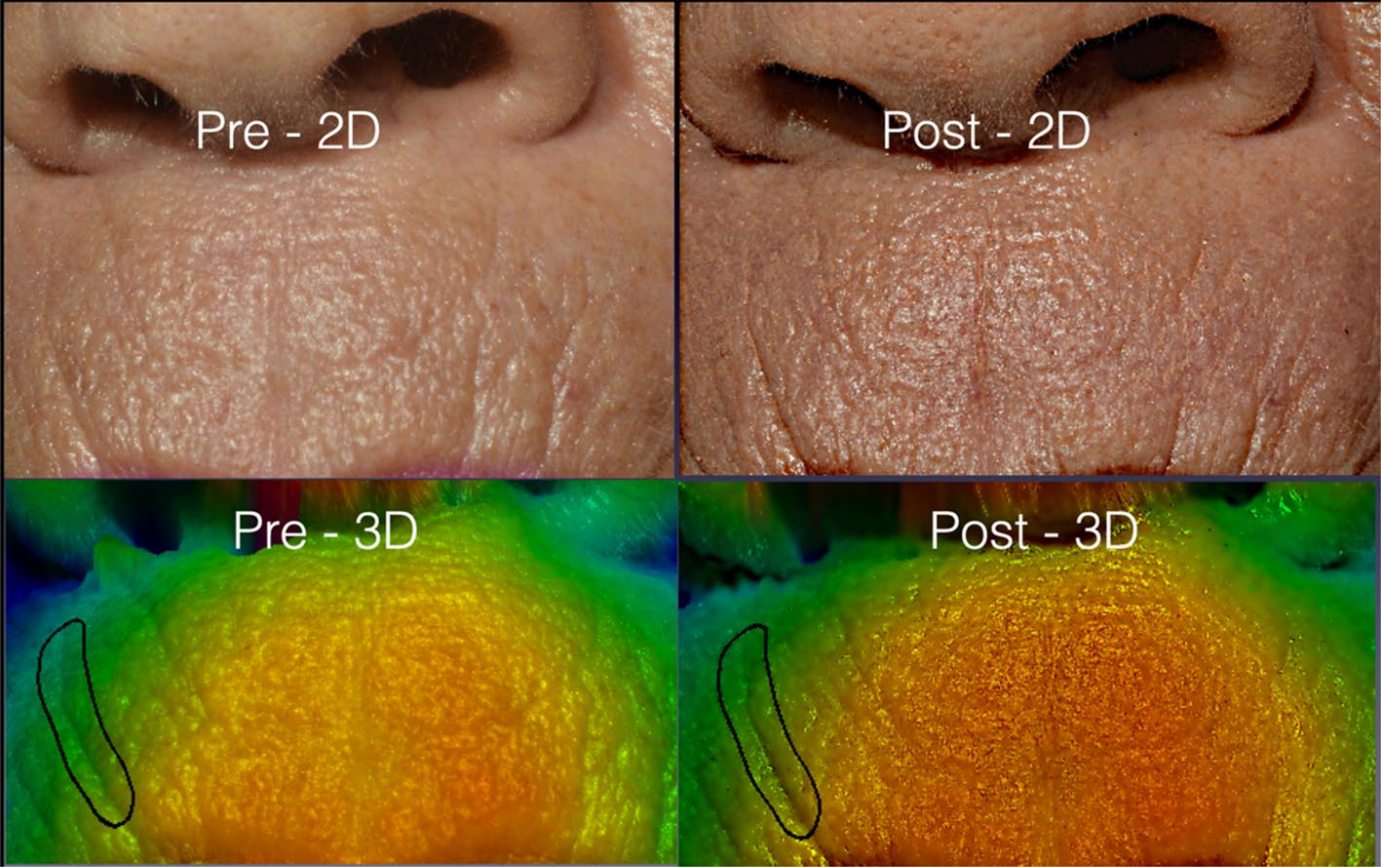
Patient randomised as the negative control. The black contour delimits a perioral wrinkle that has not been treated. The observers estimated an average of improvement up to 20% inside the designed area, whereas the 3D SPM system confirmed the absence of treatment.

The correlation of data related to the blind negative control confirmed that the observers tended to overestimate the skin improvement (Fig. 3). The raters provided scores 1/0/0 and estimated an average of 20% of improvement inside the designed area. Nonetheless, the 3D SPM confirmed that roughness remained 0.46 and that the average depth of the wrinkles kept − 0.01 mm (score 0).

**Figure 4:**
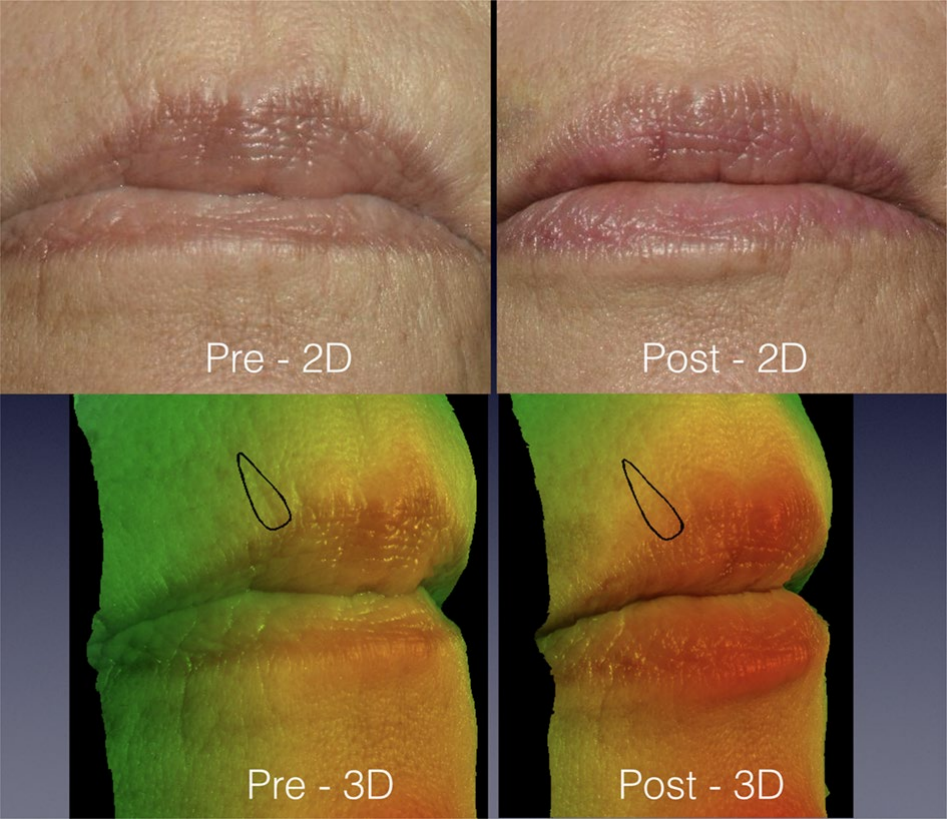
A 67-year-old patient with perioral wrinkles. The black contour specified the wrinkle to be analysed by the observers and by 3D SPM. Both methods agreed that the wrinkle demonstrated a mild improvement (< 50%).

Figure 4 displays a 67-year-old patient with perioral wrinkles. The roughness was 1.2 pre-treatment and 1.0 post-treatment whereas the average depth reduced from − 0.18 mm pre-treatment to − 0.14 mm post-treatment. The raters were unanimous by providing a score 1 regarding the wrinkles' improvement. They estimated skin improvement by 40%. The simultaneous computation of the average depth ∂ reduction (22.22%) plus the roughness ∂ reduction (16.67%) was 38.89% which confirms the agreement between both methods.

**Figure 5:**
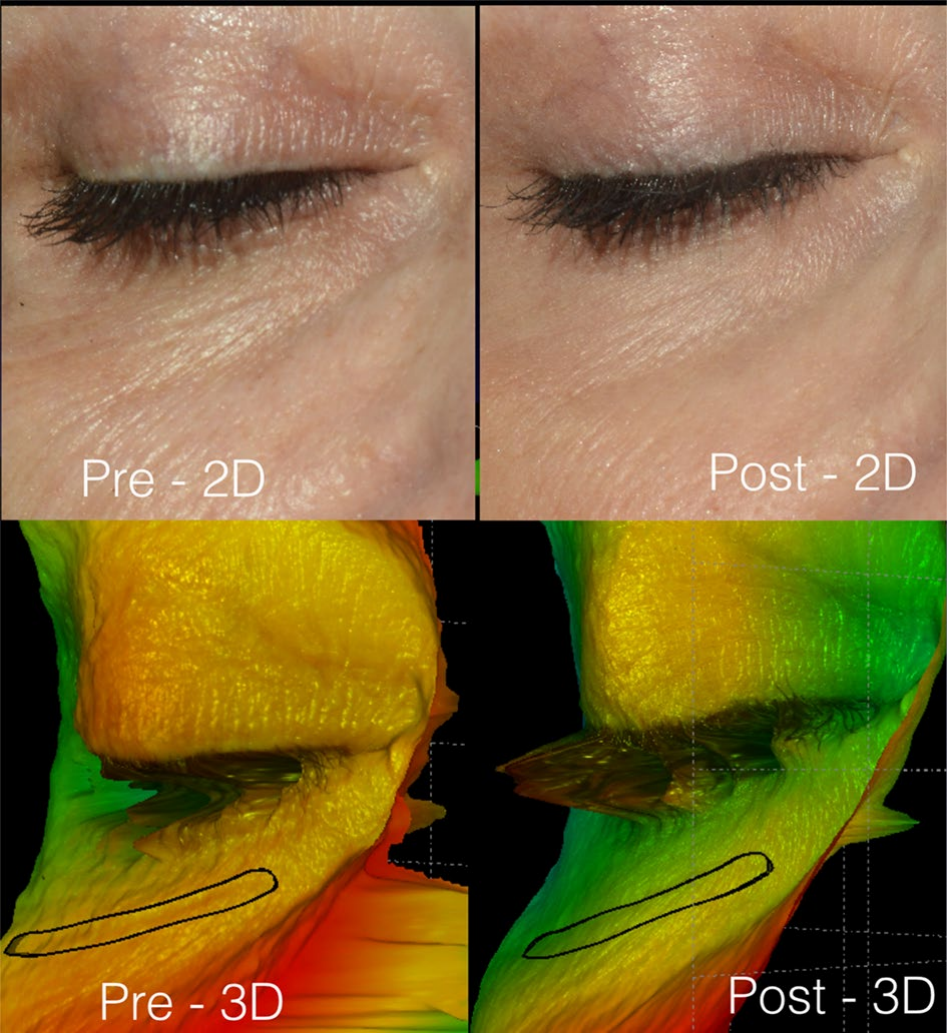
A 56-year-old patient complaining of delicate periorbital wrinkle on the right periorbital area (Fitzpatrick grade 1.5: visible wrinkle and clear indentation less than 1 mm in depth). The observers tended to overestimate the wrinkle amelioration whereas the 3D SPM system quantified it as a mild improvement.

Figure 5 exemplifies the precise measurement provided by 3D SPM software in the unassertive changes. Roughness reduced from 0.24 to 0.17 (29.16%) and the wrinkle depth diminished by 16.67% (from − 0.03 to − 0.025). The scores given by the observers varied from 1 to 3 and they estimated the skin improvement by 70%. This demonstrates the difficulty of the observers whilst judging minor cutaneous interferences based solely on photographs and reinforce the necessity of accurate methods to evaluate skin surface changes.

## Discussion

This study compares and correlates the performance of the Investigator Global Aesthetic Improvement Scale (IGAIS) and the objective data provided by a three-dimensional imaging system to investigate skin change microtopography after laser skin resurfacing followed by topical application of vitamin C. The former involves input from clinical specialists, and the latter is an objective method of computing skin change. These assessments provide different interpretations and biases, especially when being compared^4, 6, 10, 11, 43, 47^.

In 2015, Dobos et al.^4^ performed a systematic review of 111 clinical observation scales used to compare accuracy of reporting^4, 6, 11^. Their study criticised the limited evidence supporting their usage, the assessors’ innate subjectivity and the difficulty in rating minor changes on photographic imagery^4, 11, 43, 47^. Most contemporary clinical scales have been developed to analyze specific facial areas and are not applicable to other corporal areas^4, 6, 11, 44^. IGAIS has been used as an instrument to measure skin changes after skin surface treatment, independent from the anatomical region and regardless of the nature of the intervention.

The camera was reported to present an average depth precision of 0.008 mm (8 μm), and the average depth error is 0.066 mm (66 μm) for a measurement surface of 178mm^2^. This precision calculation was established in previous studies and was corrected for systematic bias^31^. We observed some loss of data accruing from the software’s inability to interpret dark holes such as nostrils or areas containing hair strands, which impeded the 3D reconstruction of some images. Nonetheless, the biggest challenge was to establish the angle and central axis of the area to be scanned so that the precise overlap between pre and post-treatment photographs could be assured^5, 12, 26, 36^. The camera has been handled only by the first author, neither have we needed to test the inter-operator reliability, nor have we necessitated to establish the coefficient of variation (CV) in the use of the equipment.

Answering one of the questions, the observers considered that the 3D images improved their capacity to use IGAIS to quantify after-treatment volumetric changes. This statement has been confirmed by statistical means. The higher ICC after evaluation of the 3D images confirmed that the observers experienced greater perception of depth and volume of the deformity and that their judgement was coherent. As a statistical limitation, we observed an increase in type I error rate accruing from the numerous variables being tested for ICC.

As for the question concerning the scale sufficiency to quantify volumetric changes or skin relief alterations, the observers complained that the scale restricted their evaluation to zero or positive values. Negative outcomes were detected only by 3D SPM because the quartile scale did not include values under zero^4, 6, 11^. This reduced the correlation between both methods for cases of an adverse outcome, because only the software was capable of detecting and quantifying any negative change presenting as a worsening of the skin condition.

Overall, the percentage of improvement scored by the raters matched the 3D SPM findings and the null hypothesis that the methods would not agree was rejected. The inter-rater agreement measured by correlation tests (Spearman’s *rho*) was higher when the ratings provided by each observer was based on 3D images.

As a limitation, the use of a four-point visual analogue scale (IGAIS) might have reduced the statistical concordance between the ordinal data provided by both methods because of the broad range between each score. Despite the perfect concordance between the scores provided by observer A and the 3D SPM system, the scores given by raters to the negative control and to the negative outcomes ratified that people, whether laic or professional, perceive the severity of the deformities differently.

In agreement with other authors, the 3D SPM system has accumulated consistent, precise and meaningful information concerning treatment-related morphologic changes and enabled a comparison of outcome^3, 16, 25, 36^.

## Conclusion

The goal of the present study was to investigate the accuracy and potential of two different methods in assessing skin modification on specific cutaneous areas: the clinical scale (IGAIS) and a three-dimensional imaging system. In particular, these methods were used to assess changes in skin microtopography after laser-assisted topical vitamin C medication.

The presence of pigmentation or variable characteristics of scar and wrinkles did not affect the observers’ nor the software’s capability to provide meaningful data. The agreement between both methods was higher when comparing the 3D images and ratings by scores. The statistical tests confirmed that the human eye perceived the most meaningful alteration; this was also detected by the software. However, adverse outcomes and cases involving subtle results were better, if not only, registered by the software. Divergent results were found in the negative control case and with adverse outcomes. The blind negative control misguided the raters whilst the numerical data provided by 3D SPM was consistent with the absence of intervention.

Three-dimensional stereophotogrammetry eliminates any potential bias or observer inconsistency because it is a more objective analysis and delivers accurate information by measuring geometric and volumetric changes in response to surface skin treatment.

## Data Availability

Raw data, additional tables and graphics not included in this version are available for consultation.
